# Asymptomatic and Mild SARS-CoV-2 Infections Elicit Lower Immune Activation and Higher Specific Neutralizing Antibodies in Children Than in Adults

**DOI:** 10.3389/fimmu.2021.741796

**Published:** 2021-09-30

**Authors:** Maria Raffaella Petrara, Francesco Bonfante, Paola Costenaro, Anna Cantarutti, Francesco Carmona, Elena Ruffoni, Costanza Di Chiara, Marisa Zanchetta, Luisa Barzon, Daniele Donà, Liviana Da Dalt, Alessio Bortolami, Matteo Pagliari, Mario Plebani, Paolo Rossi, Nicola Cotugno, Paolo Palma, Carlo Giaquinto, Anita De Rossi

**Affiliations:** ^1^ Oncology and Immunology Section, Department of Surgery, Oncology and Gastroenterology, University of Padova, Padova, Italy; ^2^ Department of Comparative Biomedical Sciences, Istituto Zooprofilattico Sperimentale delle Venezie (IZSVe), Virology Laboratory, Legnaro, Italy; ^3^ Division of Pediatric Infectious Diseases, Department for Women’s and Children’s Health, University of Padova, Padova, Italy; ^4^ Laboratory of Healthcare Research and Pharmacoepidemiology, Department of Statistics and Quantitative Methods, University of Milano-Bicocca, Milano, Italy; ^5^ Immunology and Diagnostic Molecular Oncology Unit, Veneto Institute of Oncology IOV-IRCCS, Padova, Italy; ^6^ Department of Molecular Medicine, University of Padova, Padova, Italy; ^7^ Department of Laboratory Medicine, University-Hospital of Padova, Padova, Italy; ^8^ Department of Medicine-DIMED, University of Padova, Padova, Italy; ^9^ Clinical and Research Unit of Clinical Immunology and Vaccinology, Bambino Gesù Children’s Hospital, IRCCS, Rome, Italy; ^10^ Chair of Pediatrics, Department of Systems Medicine, University of Rome “Tor Vergata”, Roma, Italy

**Keywords:** Tregs and Bregs, immune activation, senescence, COVID-19 children, neutralizing antibodies (NAbs)

## Abstract

**Background:**

The immune response plays a pivotal role in dictating the clinical outcome in severe acute respiratory syndrome coronavirus 2 (SARS*-*CoV*-*2)-infected adults, but it is still poorly investigated in the pediatric population.

**Methods:**

Of 209 enrolled subjects, 155 patients were confirmed by PCR and/or serology as having coronavirus disease 2019 (COVID-19). Blood samples were obtained at a median of 2.8 (interquartile, 2.1–3.7) and 6.1 (5.3–7.2) months after baseline (symptom onset and/or first positive virus detection). The immune profiles of activation, senescence, exhaustion, and regulatory cells were analyzed by flow cytometry. Neutralizing antibodies (nAbs) were detected by a plaque reduction neutralization test. In available nasopharyngeal swabs at baseline, SARS-CoV-2 levels were quantified by digital droplet PCR (ddPCR).

**Results:**

Overall, COVID-19 patients had higher levels of immune activation, exhaustion, and regulatory cells compared to non-COVID-19 subjects. Within the COVID-19 group, activated and senescent cells were higher in adults than in children and inversely correlated with the nAbs levels. Conversely, Tregs and Bregs regulatory cells were higher in COVID-19 children compared to adults and positively correlated with nAbs. Higher immune activation still persisted in adults after 6 months of infection, while children maintained higher levels of regulatory cells. SARS-CoV-2 levels did not differ among age classes.

**Conclusions:**

Adults displayed higher immune activation and lower production of anti-SARS-CoV-2 nAbs than children. The different immune response was not related to different viral load. The higher expression of regulatory cells in children may contribute to reduce the immune activation, thus leading to a greater specific response against the virus.

## Introduction

Severe acute respiratory syndrome coronavirus 2 (SARS-CoV-2), the causative agent of coronavirus disease (COVID)-19, emerged in China in late December 2019 and subsequently spread globally. SARS-CoV-2 has affected children less severely than adults (1) because a large majority of children usually present with asymptomatic or paucisymptomatic outcomes (2), and only a minority develop severe/critical COVID-19 and/or a COVID-19-related multisystem inflammatory syndrome (MIS-C) (3). It was first hypothesized that children had a milder disease because of the lower expression levels of the angiotensin-converting enzyme 2 (ACE-2) receptor, and thus lower viral load than adults (1), but so far, there is no evidence of a lower degree of tissue expression or function of ACE-2 in children (4), and emerging data suggest that viral loads do not differ significantly between young and old age groups (5–7).

The immune response plays a pivotal role in dictating the clinical outcome in SARS-CoV-2-infected patients. Nonetheless, while a large number of studies have been conducted in adults, the disease has been poorly investigated in the pediatric population. In adults, SARS-CoV-2 infection induces spike-specific neutralizing antibodies (8) and a specific response *via* T cells (9). However, high levels of immune activation and an overproduction of proinflammatory cytokines have been consistently described in SARS-CoV-2-infected adults, and this pattern has been associated with the severe clinical outcome of COVID-19 (10–12).

To date, very little is known about the immunopathogenesis of pediatric COVID-19. In asymptomatic/mildly symptomatic children, peripheral blood lymphocytes remain mostly in the normal range, suggesting less immune dysfunction (13, 14), but few data are available concerning their specific immune response against the virus (15) and their status of immune activation and cytokines storm (16). Children have yet to be included in clinical trials of the COVID-19 vaccine, thus understanding the immunopathogenesis of COVID-19 may provide important clues for effective treatments of this disease and the best strategy to fight infection in the pediatric population.

In this study, we studied the immune profiles of activation, senescence, exhaustion, and regulatory cells, and we analyzed their relationship with neutralizing antibodies and viral load in asymptomatic and mild symptomatic COVID-19 children and adults belonging to the same family cluster.

## Materials and Methods

### Study Population and Sampling

A single-center, prospective study was conducted on Italian family clusters of COVID-19 attending the COVID-19 Family Cluster Follow-up Clinic (CovFC), at the Department of Women’s and Children’s Health of the University Hospital of Padova (Veneto Region, Italy). From March 1 to September 4, 2020, 57 families were enrolled who met the following inclusion criteria: (a) having children of pediatric age (<15 years) and/or (b) any family member with an history of confirmed COVID-19. Families were enrolled 4–8 weeks after the end of either isolation or hospitalization and after referral from family pediatricians. Evaluation of children and relatives included data collection on demographic parameters and medical history. Parents or legally authorized representatives were informed of the research proposal and provided written consent for the collection and use of biological specimens and routine patient-based data for research purposes. The protocol was communicated to the Ethical Committee according to the national regulation (Prot. No. 0070714 of November 24, 2020; amendment no. 71779 of November 26, 2020).

A total of 209 family members were enrolled (**Supplementary Figure S1**). One hundred fifty-five subjects were considered confirmed COVID-19 cases if they had a record of virological positivity for SARS-CoV-2 by real-time PCR and/or resulted positive by either of the two serological tests adopted in the study (CLIA) MAGLUMI™ 2019-nCoV IgM/IgG and/or by plaque reduction neutralizing test (PRNT) (17). For each confirmed COVID-19 case, the baseline date was defined as follows: (1) for symptomatic case, the date of the onset of symptoms or the date of first positive SARS-CoV-2 molecular assay; (2) for asymptomatic cases, the date of the first positive molecular assay or, in those with only serologically confirmed COVID-19 and with negative/undetermined nasopharyngeal swab (NPS), by the family outbreak temporal sequence, coinciding with the date of symptoms onset in the family cluster. Fifty-four subjects that were asymptomatic and had no analytical evidence of SARS-CoV-2 infection were considered non-COVID-19 cases.

For all enrolled family members, a blood sample was collected in ethylenediaminetetraacetic acid (EDTA)-containing tube at median of 2.8 [interquartile (IQR), 2.1–3.7] months after baseline and for 116 members a follow-up sample at 6.1 (5.3–7.2) months after baseline. Plasma and cells were separate by Ficoll–Paque gradient (Pharmacia, Uppsala, Sweden). Plasma was collected, centrifuged, and appropriately stored at −80°C until use. Cells were appropriately stored at liquid nitrogen.

### SARS-CoV-2 Viral Load Quantification

A selection of 41 NPS of enrolled subjects, collected at a median of 3 (1–5) days after symptoms and originally screened at University Hospital of Padova, was made available in order to quantify the viral load. Levels of SARS-CoV-2 were quantified using a home-made multiplex quantitative assay based on One-Step RT digital droplet PCR (ddPCR) (15, 17). Results were expressed as SARS-CoV-2 copies/5 µl.

### Flow Cytometry

Cells were thawed, washed, and stained for 20 min in the dark with the Live/Dead Fixable Near-IR Dead Cell Stain Kit (Life Technologies, Carlsbad, CA, USA) and the following labeled monoclonal antibodies: anti-CD3 [fluorescein isothiocyanate (FITC)], anti-CD4 [peridinin chlorophyll protein (PerCP)], anti-CD38 [phycoerythrin (PE)], anti-HLA-DR [allophycocyanin (APC)], anti-CD279 (programmed cell death 1, PD-1) (PE-Cy7), anti-CD57 (PE), anti-CD21 (BV421), anti-CD27 (PE-Cy7), and anti-IgD (PE) (Becton-Dickinson, San Diego, CA, USA); anti-CD8 (VioGreen), anti-CD28 (APC), anti-CD19 (VioBright515), and anti-CD10 (APC) (Miltenyi Biotec, Auburn, California USA). Cells were then washed and resuspended in phosphate-buffered saline (PBS) supplemented with 1% paraformaldehyde. Tregs were determined using anti-CD4 (BB515), anti-CD25 (BV421), anti-CD127 (PE-CF594) (Becton-Dickinson, San Diego, CA, USA), and combined membrane and intracytoplasmic staining for anti-FoxP3 (AlexaFluor 647) using a transcription factor buffer set according to the manufacturer’s protocol (Becton-Dickinson, San Diego, CA, USA). All samples were analyzed using an LSRII flow cytometer (Becton-Dickinson, San Diego, CA, USA). A total of 50,000 events were collected in the lymphocyte gate using morphological parameters (forward and side-scatter). Data were processed with FACSDiva Software (Becton-Dickinson) and analyzed using Kaluza Analyzing Software v.1.2 (Beckman Coulter) (**Supplementary Figure S2**).

### Circulating Levels of PAMPS, DAMPS, and Cytokines

DNA was extracted from 200 µl of plasma using the QIAamp DNA Mini Kit (QIAGEN, Hilden, Germany) and eluted in 50 µl of AE buffer. To quantify circulating levels of 16S ribosomal (r)DNA and mitochondrial (mt)DNA, two quantitative methods based on real-time PCR assay were performed with primer pair and probe as previously described (18). Results were expressed as 16S rDNA copies/μl plasma and as mtDNA copies/μl plasma.

Plasma samples were thawed at room temperature and circulating levels of interleukin (IL)-6, IL-10, and tumor necrosis factor (TNF)-α were quantified with Fluorokine MAP Human IL-6 kit, Fluorokine MAP Human IL-10 kit (R&D Systems), and Fluorokine MAP Human TNF-α/TNFSF2 kit designed for using the Luminex 200TM according to the manufacturer’s instructions (18).

### Statistical Analysis

The immune response was assessed by comparing the median (IQR) of viral load, levels of PRNT values, and the proportion of immune activation, senescence, exhaustion, and regulatory cells, in the overall dataset, and stratified by age classes (age <6 years, 6≤ age <15 years, and age ≥15 years) between COVID-19 infected and non-infected patients. Comparisons were made by the Wilcoxon rank sum test and Kruskal–Wallis test, as appropriate.

Strength of associations between the immunology response and antibody titers, the time between first serological sampling and baseline, age, and the viral load (where possible) among infected patients was assessed by Spearman’s correlation coefficients overall and stratified by age classes.

A linear-log regression model was used to assess the association between the immunology response, and the infection, using the logarithm transformation given data skew, adjusting by age. While a log–log model was used to assess the association between the immunology response and the infection, adjusting by age and time between first serological sampling and baseline.

Finally, among 116 subjects who recorded the second peripheral blood sample, a dependent non-parametric Wilcoxon signed-rank test for subject-paired samples was used to compare the median and IQR among age classes.

Analyses were performed using the Statistical Analysis System software (version 9.4; SAS Institute, Cary, NC, USA). Statistical significance was set at the 0.05 level. All p values were two-sided.

## Results

### Patients’ Characteristics

Descriptive characteristics of patients are shown in **Table 1**. In total, 152 confirmed COVID-19 cases were studied: 70 children/older siblings and 82 parents with a median age of 8.0 (4.3–12.6) and 41.7 (33.5–46.5) years, respectively. In addition, 54 non-COVID-19 cases were studied as controls: 30 children/siblings and 24 parents, with a median age of 5.4 (3.2–8.8) and 42.1 (38.7–45.3) years, respectively. Most of COVID-19 children (75.7%) and adults (79.3%) were mild symptomatic, according to WHO guidelines (19). To better evaluate the immunological profile, the cohort was further stratified based on both social and biological development into children of pediatric age [<6 years (preschool children) and 6≤ age <15 years school age, but still pediatric subjects] and sexually mature subjects (≥15–60 years, defined as adults) (17).

**Table 1:** Descriptive analysis of the 57 families studied at the Department of Women’s and Children’s Health of the University Hospital of Padova (Italy).

	Overall			Children/older siblings			Parents		
	Non-COVID-19	COVID-19	*p-value* ^§^	Non-COVID-19	COVID-19	*p-value* ^§^	Non-COVID-19	COVID-19	*p-value* ^§^
	(n = 54)	(n = 152)		(n = 30)	(n = 70)		(n = 24)	(n = 82)	
**Female** n (%)	23 (42.6%)	78 (51.3%)	0.27	12 (40%)	33 (47.1%)	0.51	11 (45.8%)	45 (54.9%)	0.44
**Age** Median (IQR)	15.6 (4.5–41.6)	28.6 (8.2–42.1)	0.33	5.4 (3.2–8.8)	8 (4.3–12.6)	0.21	42.1 (38.7–45.3)	41.7 (33.5–46.5)	0.13
**Age classes** n (%)									
<6 years	15 (27.8%)	27 (17.8%)	0.24	15 (50%)	27 (38.6%)	0.52	–	–	
6≤ age <15	12 (22.2%)	32 (21.1%)		12 (40%)	32 (45.7%)		–	–	
≥15 years	27 (50%)	93 (61.2%)		3 (10%)	11 (15.7%)		24 (100%)	82 (100%)	
WHO classification* n (%)									
Asymptomatic	–	25 (16.5%)		–	16 (22.9%)		–	10 (12.2%)	
Mild	–	119 (78.3%)		–	53 (75.7%)		–	65 (79.3%)	
Moderate	–	6 (4%)		–	1 (1.4%)		–	5 (6.1%)	
Severe	–	1 (0.7%)		–	0 (0%)		–	1 (1.2%)	
Critical	–	1 (0.7%)		–	0 (0%)		–	1 (1.2%)	
**Pediatric comorbidities** n (%)									
No	–	–		27 (90%)	54 (77.1%)	0.13	–	–	
Yes**	–	–		3 (10%)	16 (22.9%)		–	–	

^§^Student’s t-test, χ^2^ test, Fisher exact test where appropriate.

*WHO, World Health Organization.

**The following co-morbidities were found among 16 COVID-19 positive children: premature birth (n = 1), asthma (n = 5), allergy (n = 1), congenital heart disease (n = 1), rheumatic disease (n = 1), chronic neuropathy (n = 1), immune deficiency (n = 2), cleft lip and palate (n = 1), and kidney/ureteral disease (n = 1).

### Immunological Profile: Comparison Between COVID-19 and Non-COVID-19 Subjects and Between Age Classes at First Sampling

For all enrolled subjects, a first peripheral blood sample was available at a median of 2.8 (2.1–3.7) months after baseline. Differences in the immunological parameters among COVID-19 and non-COVID-19 cases were explored by a univariate linear-log regression model and a multivariate model adjusted by age (**Supplementary Table S1**): the two groups differed in their percentages of both T (CD4 and CD8) and B-activated cells and Tregs and Bregs (**Supplementary Table S1**). Furthermore, COVID-19 adults showed higher percentages of senescent CD4 and CD8 cells and CD8 exhausted cells compared to non-COVID-19 adults, while no significant differences occurred between COVID-19 and non-COVID-19 children (**Supplementary Table S2**).

**Figure 1:**
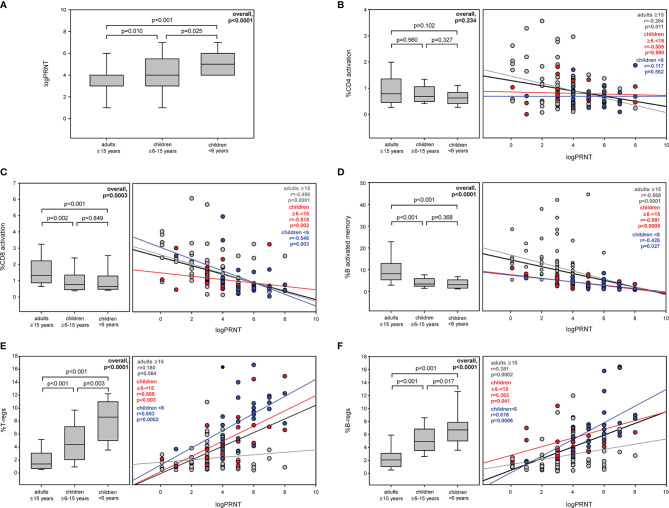
Levels and relationship between immune activation and Tregs and Bregs with PRNT at first sampling Levels of PRNT **(A)**, activated CD4 **(B)**, activated CD8 **(C)** and activated memory B cells **(D)**, Tregs **(E)**, and Bregs **(F)** in COVID-19 subjects among age classes and the relationship of these immunological markers with PRNT values at 2.8 (2.1–3.7) months after baseline.

Within the COVID-19 group, the percentage of activated cells was higher in adults than in children aged 6–15 and <6 years (**Table 2** and **Figures 1B–D**). Moreover, COVID-19 adults had higher levels of immune senescent T and B cells compared to children, and a higher expression of CD4 and CD8 exhausted cells compared to children (**Table 2**).

**Table 2:** Immunological parameters in COVID-19 age classes at first sampling.

	Children <6 years	Children 6≤ age <15 years	Adult ≥ 15 years	
Immunological parameters	(N = 27)	(N = 32)	(N = 93)	Overall p-value*
	Median (IQR)	Median (IQR)	Median (IQR)	
**%CD4 activation** **(CD4+HLA-DR+CD38+)**	0.63 (0.39–0.87)	0.58 (0.45–1.06)	0.78 (0.46–1.33)	0.234
**%CD8 activation** **(CD8+HLA-DR+CD38+)**	0.68 (0.47–1.44)	0.87 (0.51–1.32)	1.26 (0.86–2.2)	**0.0003**
**%B activated memory** **(CD19+CD10−CD21−CD27+)**	3.04 (1.51–5.17)	3.86 (2.48–5.83)	8.06 (4.89–12.63)	**<0.0001**
**%CD4 senescence** **(CD4+CD28−CD57+)**	0.56 (0.28–2.7)	0.64 (0.41–2.33)	2.14 (0.89–6.17)	**<0.0001**
**%CD8 senescence** **(CD8+CD28−CD57+)**	3.85 (1.77–7.75)	8.44 (4.06–13.54)	13.04 (10.44–20.38)	**<0.0001**
**%B senescence** **(CD19+IgD−CD27−)**	7.83 (5.04–15.42)	15.27 (8.73–16.89)	16.13 (11.8–21.6)	**<0.0001**
**%CD4 exhaustion** **(CD4+PD-1+)**	7.83 (3.43–16.17)	10.27 (5.61–15.17)	12.63 (6.7–20.57)	0.112
**%CD8 exhaustion** **(CD8+PD-1+)**	8.85 (5.82–13.28)	9.73 (6.63–16.56)	13.67 (8.63–20.98)	**0.014**
**%T-regs** **(CD4+CD25+CD127−FoxP3+)**	8.29 (4.66-10.79)	4.51 (2.66-7.22)	1.42 (0.71-3.3)	**<0.0001**
**%B-regs** **(CD19+CD24hiCD38hi)**	6.70 (4.79-8.43)	4.83 (3.52-6.76)	2.11 (1.06-3.14)	**<0.0001**
**logPRNT**	5 (4-6)	4 (3-5.5)	3 (3-4)	**<0.0001**

*Kruskal–Wallis test. Bold values refer to statistically significant p-values.

The entire COVID-19 group had a greater expression of Tregs and Bregs cells than the entire non-COVID-19 group (**Supplementary Table S1**), and this was confirmed when analysis was performed in age subgroup (**Supplementary Table S2**). Notably, within the COVID-19 subjects, children <6 years had a higher expression of Tregs and Bregs when compared with COVID-19 children 6–15 years and adults (**Table 2**).

### PRNT and Relationship With Immune Profile Among COVID-19 Subjects at First Sampling

COVID-19 children <6 years had the highest titer of nAbs [5(4–6) vs. 4(3–6) vs. 3(3–4) logPRNT, overall, p < 0.0001] (**Figure 1A**) (17).

Overall, immune activation inversely correlated with PRNT titer (%CD4+HLA-DR+CD38+: r = −0.201, p = 0.013; %CD8+HLA-DR+CD38+: r = −0.563, p < 0.0001; %CD19+CD10−CD21−CD27+: r = −0.636, p < 0.0001) (**Supplementary Table S3**). This inverse relationship was confirmed in all age groups, and adults had the greatest negative association (**Figures 1B–D**). In addition, senescent CD4, CD8, and B cells were inversely associated with PRNT titer (%CD4+CD28−CD57+: r = −0.097, p = 0.237; %CD8+CD28−CD57+: r = −0.220, p = 0.006; %CD19+IgD−CD27− r = −0.309, p = 0.0001). Stratifying by age, in adults, PRNT inversely correlated with B-cell senescence (r = −0.222, p = 0.032), while in children 6–15 years, a mild negative correlation was found between CD4 and CD8 senescent cells and PRNT titer (%CD4+CD28−CD57+: r = −0.325, p = 0.069; %CD8+CD28−CD57+: r = -0.353, p = 0.047) (**Supplementary Table S3**). No correlation was found between CD4 and CD8 exhausted cells and PRNT (%CD4+PD−1+: r = 0.035, p = 0.669; %CD8+PD-1+: r = −0.025, p = 0.756). However, in adults, there was a positive relationship between PRNT and expression of PD-1 in CD4 and CD8 (%CD4+PD-1+: r = 0.256, p = 0.013; %CD8+PD-1+: r = 0.209, p = 0.045), no relationship was found in children (**Supplementary Table S3**).

Overall, Tregs and Bregs cells positively correlated with PRNT titer (%CD4+CD25+CD127−FoxP3+: r = 0.488, p < 0.0001, %CD19+CD24hiCD38hi: r = 0.548, p < 0.0001). Notably, this positive correlation was confirmed in children (**Figures 1E, F** **)**, while in adults, there was no correlation between Tregs and log PRNT (**Figures 1E, F** **)**.

### Circulating Markers of Inflammation: PAMPs, DAMPs, and Cytokines at First Sampling

**Figure 2:**
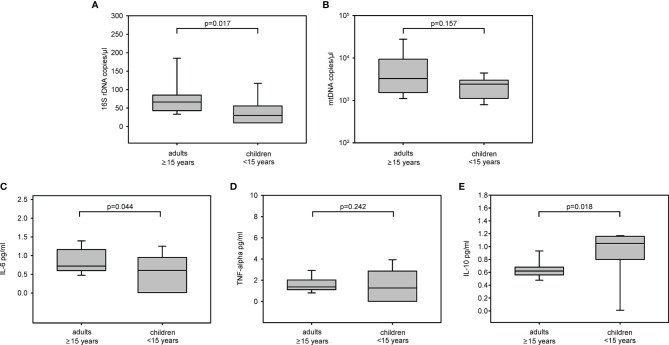
Circulating markers of PAMPs, DAMPs, and inflammatory cytokines in COVID-19 subjects at first sampling. 16S rDNA **(A)**, mtDNA **(B)**, IL-6 **(C)**, TNF-α **(D)** and IL-10 **(E)** levels in COVID-19 patients among age classes at 2.8 (2.1–3.7) months after baseline.

In a subgroup of 49 available plasma samples from COVID-19 patients, collected at a median of 2.8 (2.1–3.7) months after baseline, circulating levels of PAMPs (16S rDNA), DAMPs (mtDNA), and cytokines (IL-6, TNF-α, and IL-10) were quantified. Given the low number of samples, the cohort was divided into adults (≥15 years, n = 29) and children (<15 years, n = 20). Adults had higher plasma levels of 16S rDNA [66 (43–86) *vs.* 32 (10–67) copies/µl, p = 0.017] and mtDNA [3,289 (1,451–10,497) *vs.* 2,553 (1,118–3,703) copies/µl, p = 0.157] than children (**Figures 2A, B** **)**. IL-6 was significantly higher in adults than children [0.8 (0.7–1.2) *vs.* 0.7 (0.1–1.0) pg/ml, p = 0.044], and TNF-α tended to be higher in adults, but not significantly [1.4 (1.1–2.4) *vs.* 1.2 (0.01–2.4) pg/ml, p = 0.242]. Conversely, IL-10 was higher in children compared to adults [1.1 (0.8–1.2) *vs.* [0.6 (0.5–0.7) pg/ml, p = 0.018) (**Figures 2C–E**).

### Viral Load and Relationship With Immune Profile

SARS-CoV-2 viral load (VL) was measured by ddPCR on available NPS. No significant differences were found among the three age groups [185,067 (326–339,315) SARS-CoV-2 copies/5 µl in adults *vs.* 6,723 (3,427–114,587) children 6–15 years old *vs.* 21,106 (162–152,500) in children <6 years old, respectively, overall, p = 0.955). The relationship among VL and immunological parameters was evaluated by stratifying the cohort into two groups: children <15 years and adults ≥15 years. Overall, only a weak negative relationship was found between VL and activated CD4 (r = −0.272, p = 0.085) and B cells (r = −0.267, p = 0.092) (**Table 3**), and in children, VL tended to be inversely correlated with B activated memory cells (r= -0.463, p=0.053) and positively correlated with Bregs (r = 0.437, p = 0.070) (**Table 3**).

**Table 3:** Relationship between viral load and immunological parameters at first sampling.

Immunological parameters	All 41 NPS		Children aged <15 years		Adults aged ≥15 years	
	R spearman	p-value^§^	R spearman	p-value^§^	R spearman	p-value^§^
**logPRNT**	0.118	0.463	**0.523**	**0.026**	0.035	0.875
**%CD4 activation** **(CD4+HLA-DR+CD38+)**	**−0.272**	**0.085**	−0.375	0.126	−0.182	0.406
**%CD8 activation** **(CD8+HLA-DR+CD38+)**	−0.083	0.607	−0.307	0.216	0.059	0.788
**%B activated memory** **(CD19+CD10−CD21−CD27+)**	**−0.267**	**0.092**	**−0.463**	**0.053**	−0.202	0.356
**%CD4 senescence** **(CD4+CD28−CD57+)**	0.077	0.638	0.197	0.433	−0.023	0.919
**%CD8 senescence** **(CD8+CD28−CD57+)**	0.159	0.321	−0.141	0.576	0.334	0.119
**%B senescence** **(CD19+IgD−CD27−)**	−0.173	0.279	−0.358	0.145	−0.197	0.369
**%CD4 exhaustion** **(CD4+PD-1+)**	−0.016	0.919	−0.216	0.390	0.076	0.730
**%CD8 exhaustion** **(CD8+PD-1+)**	0.159	0.321	−0.222	0.376	0.289	0.180
**%T-regs** **(CD4+CD25+CD127−FoxP3+)**	0.087	0.589	0.271	0.276	0.026	0.906
**%B-regs** **(CD19+CD24hiCD38hi)**	−0.053	0.744	**0.437**	**0.070**	−0.154	0.483

^§^Spearman correlation. Bold values refer to R spearman and p-values that are or tended to be significant.

### Differences of Immunological Profile and Relationship With PRNT at Second Sampling

**Figure 3:**
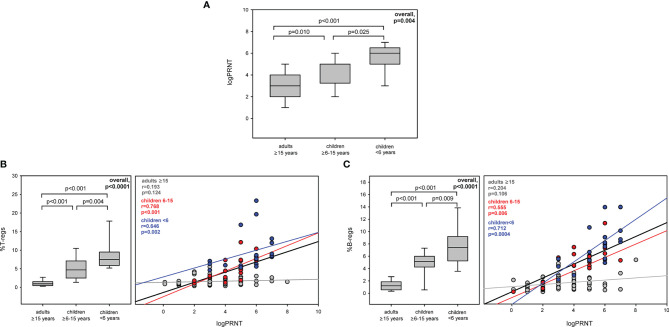
Levels and relationship between Tregs and Bregs with PRNT at second sampling. Levels of PRNT **(A)**, Tregs **(B)**, and Bregs **(C)** in COVID-19 subjects among age classes and the relationship of these immunological markers with PRNT values at 6.1 (5.3–7.2) months after baseline.

For a total of 116 subjects, a second peripheral blood sample was obtained after a median of 6.1 (5.3–7.2) months from baseline. No significant difference was seen in levels of nAbs from first and second samples in children, while they significantly decreased in adults [4 (3–4) *vs.* 3 (2–4) log PRNT, p = 0.004] (**Table 4** and **Figure 3A**). Adults still maintained a higher level of immune activation (**Table 4**), which continued to be significantly higher than those observed in children [%CD4+HLA-DR+CD38+: 0.9 (0.6–1.1) *vs.* 0.6 (0.4–1) *vs.* 0.6 (0.5–0.8), overall p = 0.132; %CD8+HLA-DR+CD38+: 1.7 (0.9–2.7) *vs.* 0.8 (0.6–1.3) *vs.* 0.7 (0.5–1.1), overall p < 0.0001; %CD19+CD10−CD21−CD27+: 9.1 (4.7–11.8) *vs.* 3.3 (2.3–4.8) *vs.* 4.2 (2.5–5.7), overall p < 0.0001]. Percentage of CD4 senescent cells increased in all age classes (**Table 4**). Notably, Tregs and Bregs significantly decreased in adults (**Table 4**), while their percentages did not change in children, and children <6 years maintained the highest expression [%CD4+CD25+CD127−FoxP3+: 7.9 (6.1–10) *vs.* 4.7 (2.4–7.1) *vs.* 1.0 (0.5–1.5), overall p < 0.0001; %CD19+CD24hiCD38hi: 7.4 (5.3–8.9) *vs.* 5.2 (4.3–5.9) *vs.* 1.2 (0.6–1.9), overall p < 0.0001]. Moreover, 6 months after infection, the positive association persisted in children between PRNT titer and Tregs (r = 0.646, p = 0.002 in children aged <6 years; r = 0.768, p < 0.0001 in children aged 6–15 years) and between PRNT titer and Bregs (r = 0.712, p = 0.0004 in children aged <6 years; and r = 0.555, p = 0.006 in children aged 6–15 years), while no relationship was found in adults (**Figures 3B, C** **)**.

**Table 4:** Differences of immunological parameters between first and second sampling.

	Children < 6 years) (n = 21)			Children 6–15 years (n = 26)			Adult > 15 years (n = 69)		
	First sample	Second sample	p-value^§^	First sample	Second sample	p-value^§^	First sample	Second sample	p-value^§^
**Median** (**IQR**) **months**	2.4 (1.9–2.8)	5.6 (4.5–5.9)		3.3 (2.6–3.8)	6.6 (5.9–7.5)		2.8 (2.1–3.6)	6.1 (5.4–7.3)	
**logPRNT**	5 (4–6)	6 (5–6.5)	0.521	5 (4–6)	5 (3–5)	0.672	4 (3–4)	3 (2–4)	**0.004**
**%CD4 activation** **(CD4+HLA-DR+CD38+)**	0.6 (0.5–0.8)	0.6 (0.5–0.8)	0.533	0.6 (0.5–1.1)	0.6 (0.4–1)	0.855	0.8 (0.4–1.3)	0.9 (0.6–1.1)	0.885
**%CD8 activation** **(CD8+HLA-DR+CD38+)**	0.7 (0.5–1.4)	0.7 (0.5–1.1)	0.750	0.8 (0.5–1.2)	0.8 (0.6–1.3)	0.618	1.2 (0.9–1.9)	1.7 (0.9–2.7)	0.058
**%B activated memory** **(CD19+CD10−CD21−CD27+)**	2.8 (1.5–4.8)	4.2 (2.5–5.7)	0.079	3.3 (2.3–4.5)	3.3 (2.3–4.8)	0.592	7.1 (4.1–11.7)	9.1 (4.7–11.8)	0.126
**%CD4 senescence** **(CD4+CD28**–**CD57+)**	0.6 (0.4–3.1)	3 (1.7–4.8)	**0.047**	0.8 (0.5–2.7)	6.5 (3.1–10)	**<0.0001**	2.5 (0.9–8.8)	6.3 (3.2–9.6)	**0.026**
**%CD8 senescence** **(CD8+CD28**–**CD57+)**	3.9 (2.0–7.8)	4.7 (2.7–7.9)	0.418	8.3 (4– 13)	8.4 (6.2–13.5)	0.956	13.8 (11–21.5)	14.7 (10.5–22.4)	0.484
**%B senescence** **(CD19+IgD−CD27−)**	10.9 (6.1–15.6)	7.8 (2.4–10.7)	0.051	14.9 (9–16.2)	13.4 (10.2–17.1)	0.303	16 (12.5–20.1)	16.5 (11.8–21.8)	0.868
**%CD4 exhaustion** **(CD4+PD-1+)**	7.7 (3.3–16.2)	7.3 (4.4–13.7)	0.927	9.2 (5.2–15.4)	10 (4.2–15.8)	0.825	13 (8.3–21.9)	11.5 (7.3–18.6)	0.430
**%CD8 exhaustion** **(CD8+PD-1+)**	9.5 (5.9–12.3)	7.8 (4.7–12.4)	0.870	9.6 (6.2–16.5)	9.9 (6.7–15)	0.719	14.2 (9.5–22)	12.2 (7.2–19.5)	0.213
**%T-regs** **(CD4+CD25+CD127-FoxP3+)**	8.3 (4.7–10)	7.9 (6.1–10)	0.498	4.5 (2.9–7)	4.7 (2.4–7.1)	0.658	1.4 (0.7–3.4)	1.0 (0.5–1.5)	**<0.0001**
**%B-regs** **(CD19+CD24hiCD38hi)**	6.1 (4.6-8.8)	7.4 (5.3–8.9)	0.468	5 (4–6.9)	5.2 (4.3–5.9)	0.310	2.1 (1.2–3)	1.2 (0.6–1.9)	**<0.0001**

^§^Wilcoxon signed-rank test. Bold values refer to statistically significant p-values.

## Discussion

Most of the studies conducted in SARS-CoV-2-infected adults confirm the pivotal relevance of immune activation and cytokine storm in dictating the clinical outcome of the infection. Most of the SARS-CoV-2-infected children are asymptomatic or very mildly symptomatic, but the immunopathogenesis of COVID-19 is still poorly investigated in the pediatric population. In the present study, for the first time, we had the opportunity to study the immune profile of SARS-CoV-2-infected adults and children, clustered within the same families, and compared to uninfected age-class matched relatives.

Immunological response to SARS-CoV-2 has been widely studied in the adult population; several studies reported that COVID-19-infected patients expressed higher percentages of activated cells and exhausted cells compared to healthy controls (11, 20). Additionally, T-cell activation and exhaustion appears to correlate with disease severity in COVID-19 patients (9, 10, 21), and the immune activation persists despite viral clearance (12, 21). Our data confirmed a higher immune activation/exhaustion in asymptomatic/mildly symptomatic COVID-19 compared to non-COVID-19 adults, and the activation still persisted after 6 months from infection.

Higher levels of activated CD4 and CD8 T cells were described in COVID-19 pediatric patients with MIS-C (22–24). COVID-19 children without or with mild/moderate clinical manifestations showed similar frequencies of activated CD4 and CD8 cells compared to age-matched control (15, 22, 25). In agreement with these findings, the present study found no differences between COVID-19 and non-COVID-19 children, and for the first time, we demonstrated that COVID-19 adults, mostly asymptomatic/mildly symptomatic, have a higher expression of both activated T and B cells, not only compared to non-COVID-19 adults but also compared to COVID-19 children.

The immune activation is a major driver of immune senescence (26, 27); the continuous cell activation and expansion of immune cells leads to their senescent status with the loss of their function (28). Consistently with this concept, COVID-19 adults had significantly higher levels of senescent T and B cells than non-COVID-19 adults. It is well known that the release of exogenous pathogen-associated molecular patterns (PAMPs) and endogenous damage-associated molecular patterns (DAMPs) into circulation through binding Toll-like receptors (TLR-9 or TLR-4) is an important driver of cytokine storm, inflammation, and immune activation (29). The significantly higher levels of PAMPs, DAMPs, and IL-6 proinflammatory cytokine in COVID-19 adults compared to COVID-19 children may contribute to explaining the higher levels of immune activation and senescence in the former. The only exception was the IL-10. Notably, activated and senescent T and B cells inversely correlated with a production of anti-SARS-CoV-2 nAbs, thus suggesting that after infection in adults, immune activation exerts a strong influence on immune aging and drains resources from the immune system for the specific production of anti-SARS-CoV-2 antibodies.

In agreement with several studies indicating that children and adults do not differ for viral load (5–7), in this study, which was conducted in asymptomatic or mild symptomatic COVID-19 patients, levels of SARS-CoV-2 at baseline did not differ among age classes. Nonetheless, the impact of viral load on adults may differ from its effect on children. It is widely reported that viral load is higher in symptomatic than asymptomatic COVID-19 adults (30). The data available in the pediatric population are controversial. Some studies found higher levels in symptomatic children compared to asymptomatic children (25, 31), while other reports found no association of viral load and disease severity (5, 32). A recent study, conducted in COVID-19 children within their first week from baseline (symptom onset and/or first positive virus detection) demonstrated an inverse relationship between viral load and nAbs, and the estimation of virus under curve from NPS, collected every 48 h up to undetectable viral load, confirmed the impact of nAbs on virus clearance (15). Our data suggested that this relationship did not persist after viral clearance.

Regulatory T cells play a crucial role in suppressing excessive immune responses to pathogens, cancer cells, and transplanted organs and in preventing and controlling the development of autoimmune and allergic diseases (33). Regulatory B cells had a negative role in immune reaction and inflammation in humans (34). Data on Tregs and COVID-19 are conflictual: COVID-19 adult patients expressed higher percentages of Tregs compared to uninfected ones (11), but decreased numbers of circulating Tregs have been described in severe COVID-19 cases (35, 36). The reduced proportion of SARS-CoV-2-reactive regulatory T cells observed in hospitalized COVID-19 patients, compared to non-hospitalized ones, suggested that a defect in the generation of immunosuppressive SARS-CoV-2-reactive Tregs was associated with a severe clinical outcome (37). No data are available for Bregs to date. In our study, both Tregs and Bregs were significantly higher in asymptomatic/mildly symptomatic COVID-19 patients compared to non-COVID-19 subjects in all age classes. Interestingly, COVID-19 children, particularly those <6 years, had higher expression of Tregs and Bregs than COVID-19 adults, and notably, this is positively associated with production of nAbs. Tregs inhibit the activation of both innate and adaptive immune response *via* inhibitory surface molecules (like CTLA-4 and LAG-3) and by the secretion of immunosuppressive cytokines (i.e., IL-10, TGF-β, and IL-35) (38, 39). It has been recently reported that slow-progressors HIV-infected children secreted higher levels of IL-10 compared to those who progressed and had higher proliferation of Tregs (40). Similarly, it is possible that Tregs and Bregs in SARS-CoV-2-infected children constrains inflammation/immune activation, likely through the release of IL-10. Indeed, a significant positive association was found between IL-10 and Tregs in children (r = 0.633, p = 0.011). Interestingly, in children, and in particular in children <6 years of age, high levels of Tregs and Bregs cells persisted for over 6 months of follow-up, and the titer of nAbs, thus supporting the concept that these cells play a role in directing the host immune response.

A limitation of this study is that it includes only asymptomatic/mildly symptomatic COVID-19 children and adults. Nonetheless, our data demonstrated that even in the absence of severe disease, COVID-19 adults showed a higher degree of hyperinflammation/immune activation than COVID-19 children, although levels of SARS-CoV-2 did not differ among classes. The immune activation, with higher release of PAMPs and DAMPs into circulation, leading to the overproduction of proinflammatory cytokine IL-6, might limit the production of anti-SARS-COV-2-neutralizing antibodies and impair the specific response in adults. Conversely, in COVID-19 children, the viral-induced inflammation may be mitigated by the higher expansion of regulatory T and B cells resulting in preserved resources for a higher specific production of anti-SARS-CoV-2-neutralizing antibodies. Further studies are needed to support the role of regulatory cells in this context.

## Data Availability Statement

The raw data supporting the conclusions of this article will be made available by the authors, without undue reservation.

## Ethics Statement

The studies involving human participants were reviewed and approved by Prot. No. 0070714 of November 24, 2020, amendment No. 71779 of November 26, 2020. Written informed consent to participate in this study was provided by the participants’ legal guardian/next of kin.

## Author Contributions

MRP, FB, PC, MP, PR, PP, CG, and ADR designed the study. MRP, FB, FC, ER, MZ, AB, and MP performed the investigations. PC, CC, LB, DD, LDD, MP, and CG provided samples and reagents. AC performed the statistical analysis. MRP wrote the manuscript. FB, PC, AC, NC, PR, PP, CG, and ADR supervised the project and reviewed and edited the manuscript. All authors contributed to the article and approved the submitted version.

## Funding

This work was supported by the EU Horizon 2020 (RECOVER) (Grant Agreement Number 101003589) and Fondazione Cassa di Risparmio di Padova e Rovigo, progetti di Ricerca COVID-19 (ADR participant).

## Conflict of Interest

The authors declare that the research was conducted in the absence of any commercial or financial relationships that could be construed as a potential conflict of interest.

## Publisher’s Note

All claims expressed in this article are solely those of the authors and do not necessarily represent those of their affiliated organizations, or those of the publisher, the editors and the reviewers. Any product that may be evaluated in this article, or claim that may be made by its manufacturer, is not guaranteed or endorsed by the publisher.
