# Supplementary material for: Asymptomatic and Mild SARS-CoV-2 Infections Elicit Lower Immune Activation and Higher Specific Neutralizing Antibodies in Children Than in Adults
Source: Front Immunol. 2021 Sep 30;12:741796. doi: 10.3389/fimmu.2021.741796 (PMC8515185; doi:10.3389/fimmu.2021.741796)
Supplement: Supplementary file 1 [file DataSheet_1.docx]

**Supplementary Figure S1.** Flow chart of family clusters of COVID-19 enrolled from March 1^st^ to the September 4^th^ 2020, at the COVID-19 follow-up clinic of the Pediatric Department, Department of Women’s and Children’s Health, University of Padua.


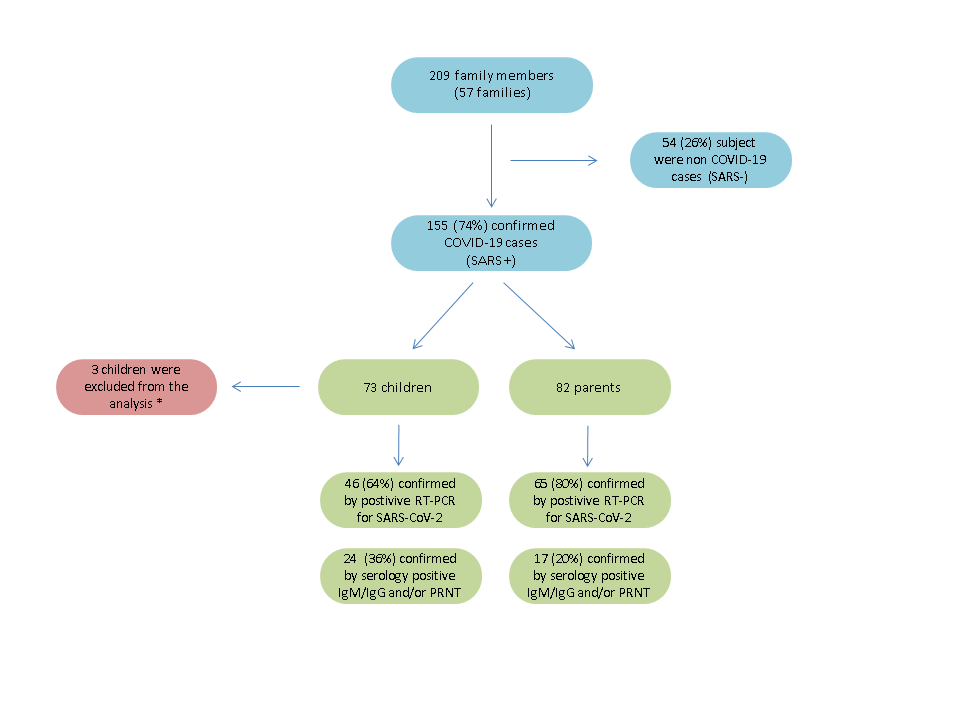


*Three children were excluded from the analysis: 2 children presented MIS-C, Multisystem Inflammatory Syndrome in Children, 4-6 weeks after COVID-19 onset and 1 newborn of a COVID-19 positive mother presented positive SARS-CoV-2 neutralizing antibodies detected 51 days after birth that could be related to maternal immunity and not seroconversion (SARS-CoV-2 molecular assay was never performed at birth).

**Supplementary Table S1.** Immunological characteristics of the overall cohort of SARS-CoV-2-infected cases at first sampling

|  | **non COVID-19** | **COVID-19** | *p-value^§^* | Linear regression model* | |
| --- | --- | --- | --- | --- | --- |
| **Immunological markers** | (N=54) | (N=152) |  |  |  |
|  | Median [IQR] | Median |  | **β** | *p-value^**^* |
| **%CD4 activation**  **(CD4+HLA-DR+CD38+)** | 0.41 [0.26-0.70] | 0.70 [0.45-1.12] | **<0.0001** | 0.45 | **0.002** |
| **%CD8 activation**  **(CD8+HLA-DR+CD38+)** | 0.82 [0.56-1.08] | 1.09 [0.68-1.82] | **0.001** | 0.25 | 0.084 |
| **%B activated memory**  **(CD19+CD10-CD21-CD27+)** | 2.16 [1.30-3.23] | 5.61 [3.15-9.93] | **<0.0001** | 1.03 | **<0.0001** |
| **%CD4 senescence**  **(CD4+CD28-CD57+)** | 0.99 [0.51-2.96] | 1.55 [0.51-4.16] | 0.211 | 0.27 | 0.217 |
| **%CD8 senescence**  **(CD8+CD28-CD57+)** | 9.64 [6.50-13.44] | 11.16 [5.79-17.86] | 0.356 | 0.00 | 0.904 |
| **%B senescence**  **(CD19-IgD-CD27-)** | 9.00 [6.20-14.51] | 15.24 [9.05-19.52] | **<.0001** | 0.50 | **0.001** |
| **%CD4 exhaustion**  **(CD4+PD-1+)** | 7.58 [4.80-11.65] | 11.75 [6.13-18.02] | **0.024** | 0.21 | 0.083 |
| **%CD8 exhaustion**  **(CD8+PD-1+)** | 8.76 [5.10-13.46] | 12.66 [6.96-19.21] | **0.013** | 0.27 | **0.018** |
| **%T-regs**  **(CD4+CD25+CD127-FoxP3+)** | 0.73 [0.39-1.99] | 2.75 [1.01-5.65] | **<0.0001** | 1.16 | **<0.0001** |
| **%B-regs**  **(CD19+CD24hiCD38hi)** | 1.69 [0.90-2.70] | 3.11 [1.69-5.75] | **<0.0001** | 0.71 | **<0.0001** |

*adjusted by age

^§^Wilcoxon rank sum test

**t-test

**Supplementary Table S2.** Immunological parameters in COVID-19 and non-COVID-19 age classes at first sampling.

|  | **children < 6 years** | | | **children 6-15 years** | | | **adult ≥ 15 years** | | |
| --- | --- | --- | --- | --- | --- | --- | --- | --- | --- |
|  | **non COVID-19** | **COVID-19** | **p-value**^§^ | **non COVID-19** | **COVID-19** | **p-value**^§^ | **non COVID-19** | **COVID-19** | **p-value**^§^ |
|  | (N=15) | (N=27) |  | (N=12) | (N=28) |  | (N=27) | (N=93) |  |
|  | Median [IQR] | Median [IQR] |  | Median [IQR] | Median [IQR] |  | Median [IQR] | Median [IQR] |  |
| **logPRNT** | 0.1 [0.1-0.1] | 5 [4-6] | **<0.0001** | 0.1 [0.1-0.1] | 4 [3-5.5] | **<0.0001** | 0.1 [0.1-0.1] | 3 [3-4] | **<0.0001** |
| **%CD4 activation**  **(CD4+HLA-DR+CD38+)** | 0.36 [0.32-0.52] | 0.63 [0.39-0.87] | 0.083 | 0.53 [0.35-0.87] | 0.58 [0.45-1.06] | 0.437 | 0.41 [0.17-0.7] | 0.78 [0.46-1.33] | **0.001** |
| **%CD8 activation**  **(CD8+HLA-DR+CD38+)** | 0.99 [0.72-1.17] | 0.68 [0.47-1.44] | 0.401 | 1.03 [0.72-1.78] | 0.87 [0.51-1.32] | 0.399 | 0.66 [0.38-0.85] | 1.26 [0.86-2.2] | **<0.0001** |
| **%B activated memory**  **(CD19+CD10-CD21-CD27+)** | 1.74 [1.29-2.92] | 3.04 [1.51-5.17] | 0.052 | 2.34 [1.17-3.25] | 3.86 [2.48-5.83] | **0.020** | 2.3 [1.3-3.25] | 8.06 [4.89-12.63] | **<0.0001** |
| **%CD4 senescence**  **(CD4+CD28-CD57+)** | 0.77 [0.17-2.48] | 0.56 [0.28-2.7] | 0.823 | 1.01 [0.7-2.27] | 0.64 [0.41-2.33] | 0.589 | 1.3 [0.68-4.38] | 2.14 [0.89-6.17] | 0.169 |
| **%CD8 senescence**  **(CD8+CD28-CD57+)** | 6.65 [4.05-9.46] | 3.85 [1.77-7.75] | 0.068 | 13.39 [9.18-18.39] | 8.44 [4.06-13.54] | 0.123 | 10.83 [8.05-13.44] | 13.04 [10.44-20.38] | **0.030** |
| **%B senescence**  **(CD19+IgD-CD27-)** | 7.08 [5.24-9.35] | 7.83 [5.04-15.42] | 0.294 | 10.49 [4.07-15.85] | 15.27 [8.73-16.89] | 0.159 | 11.76 [7.08-17.24] | 16.13 [11.8-21.6] | **0.002** |
| **%CD4 exhaustion**  **(CD4+PD-1+)** | 6.03 [3.32-9.32] | 7.83 [3.43-16.17] | 0.096 | 10.55 [6.84-15.92] | 10.27 [5.61-15.17] | 0.702 | 7.97 [4.65-14.48] | 12.63 [6.7-20.57] | 0.093 |
| **%CD8 exhaustion**  **(CD8+PD-1+)** | 4.65 [3.51-10.59] | 8.85 [5.82-13.28] | 0.088 | 11.09 [8.07-15.28] | 9.73 [6.63-16.56] | 0.802 | 9.23 [6.5-17.34] | 13.67 [8.63-20.98] | **0.039** |
| **%T-regs**  **(CD4+CD25+CD127-FoxP3+)** | 1.21 [0.96-3.03] | 8.29 [4.66-10.79] | **<0.0001** | 0.7 [0.36-1.55] | 4.51 [2.66-7.22] | **<0.0001** | 0.63 [0.31-1.17] | 1.42 [0.71-3.3] | **0.0003** |
| **%B-regs**  **(CD19+CD24hiCD38hi)** | 1.98 [0.81-2.86] | 6.7 [4.79-8.43] | **<0.0001** | 2.06 [1.18-3.07] | 4.83 [3.52-6.76] | **<0.0001** | 1.29 [0.89-2.16] | 2.11 [1.06-3.14] | **0.045** |

^§^ Wilcoxon rank sum test

**Supplementary Table S3.** Relationship between PRNT values and immunological parameters at first sampling

|  | **logPRNT** | | | | | | | |
| --- | --- | --- | --- | --- | --- | --- | --- | --- |
| **Immunological markers** | **overall,**  **n=152** | | **children <6 years n=27** | | **children 6-15 years n=28** | | **adults >15 years**  **n=93** | |
|  | R spearman | p-value^§^ | R spearman | p-value^§^ | R spearman | p-value^§^ | R spearman | p-value^§^ |
| **%CD4 activation**  **(CD4+HLA-DR+CD38+)** | -0.201 | 0.013 | -0.117 | 0.562 | -0.005 | 0.980 | **-0.264** | **0.011** |
| **%CD8 activation**  **(CD8+HLA-DR+CD38+)** | **-0.563** | **<.0001** | **-0.546** | **0.003** | **-0.519** | **0.002** | **-0.496** | **<.0001** |
| **%B activated memory**  **(CD19+CD10-CD21-CD27+)** | **-0.636** | **<.0001** | **-0.425** | **0.027** | **-0.581** | **0.001** | **-0.568** | **<.0001** |
| **%CD4 senescence**  **(CD4+CD28-CD57+)** | -0.097 | 0.237 | 0.099 | 0.625 | -0.325 | 0.069 | **0.209** | **0.046** |
| **%CD8 senescence**  **(CD8+CD28-CD57+)** | **-0.220** | **0.006** | 0.020 | 0.922 | **-0.353** | **0.047** | 0.161 | 0.122 |
| **%B senescence**  **(CD19-IgD-CD27-)** | **-0.309** | **0.0001** | -0.363 | 0.063 | -0.116 | 0.526 | **-0.222** | **0.032** |
| **%CD4 exhaustion**  **(CD4+PD-1+)** | 0.035 | 0.669 | -0,0116 | 0.954 | -0.125 | 0.494 | **0.256** | **0.013** |
| **%CD8 exhaustion**  **(CD8+PD-1+)** | -0.025 | 0.756 | -0,12573 | 0.532 | -0.042 | 0.827 | **0.209** | **0.045** |
| **%T-regs**  **(CD4+CD25+CD127-FoxP3+)** | **0.488** | **<0.0001** | **0.653** | **0.0002** | **0.505** | **0.003** | 0.180 | 0.084 |
| **%B-regs**  **(CD19+CD24hiCD38hi)** | **0.548** | **<0.0001** | **0.616** | **0.001** | **0.363** | **0.041** | **0.381** | **0.0002** |

^§^Spearman correlation

**Supplementary Figure S2. Gating strategy.** A) Flow cytometry gating strategy for CD4+ and CD8+ T cell activation (HLA-DR+CD38+), senescence (CD28-CD57+), exhaustion (PD-1+) and Tregs (CD4+CD25+CD127-FoxP3+). (B) Flow cytometry gating strategy for B cell activation (CD19+CD10-CD27+CD21-), senescence (CD19+IgD-CD27-) and Bregs (CD19+CD24hiCD38hi)**.**

**A)**

**
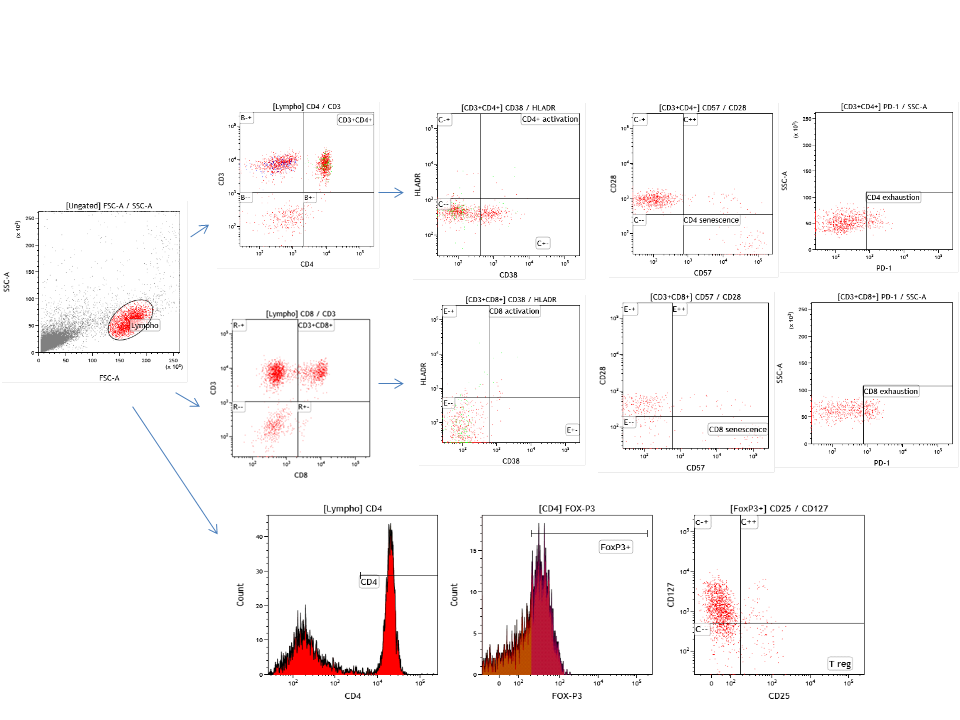
**

**B)**

**
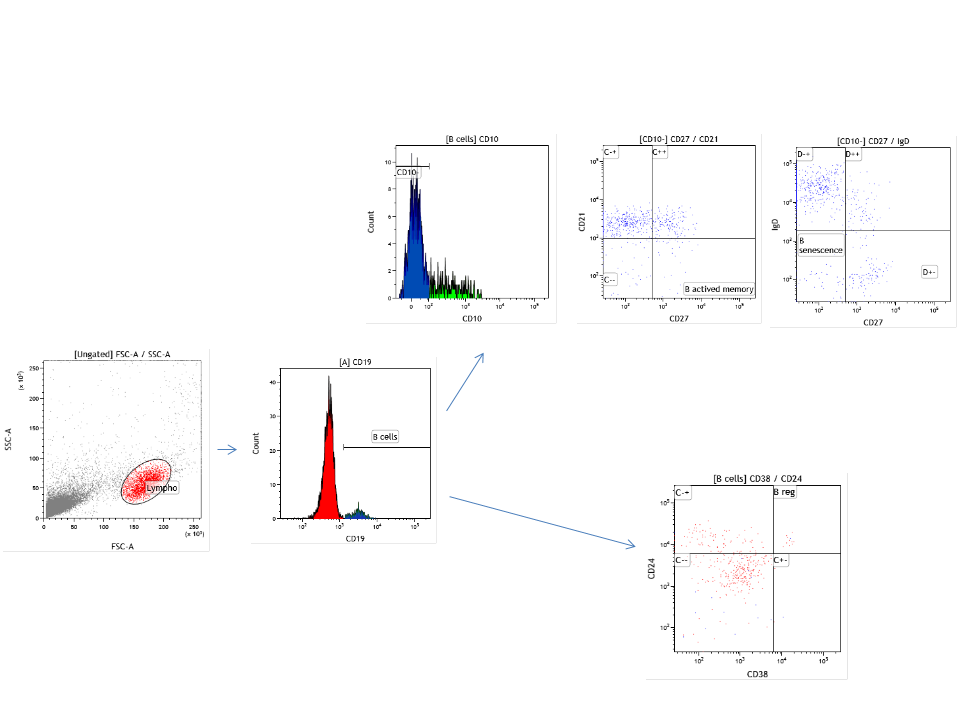
**
